# Precocious puberty and ovarian tumors – 2 case reports

**DOI:** 10.1186/1687-9856-2013-S1-P80

**Published:** 2013-10-03

**Authors:** S Ramkumar, Shivprakash, AC Ammini

**Affiliations:** 1 Department of Endocrinology and Metabolism, All India Institute of Medical Sciences, New Delhi, India

## Aim

To report 2 cases of peripheral precocious puberty due to ovarian tumors of different etiology.

## Methods

We describe the clinical presentation, imaging findings, hormonal work-up and follow up after oophorectomy of 2 children with precocious puberty due to ovarian tumors.

## Results

### Case 1

3 years old girl presented with progressive bilateral breast enlargement over 1 year, irregular vaginal bleeding for 10 months and pubic hair development for 2 months. Her past history was unremarkable. Her general examination and vitals were normal. Tanners staging was B3P3. There was no axillary hair or genital ambiguity. Cardiac, thoracic and neurological examinations were normal. A solitary well defined pelvic mass firm to hard, mobile was palpable in right lower abdomen. Her bone age, height age and chorological age were respectively 8yr 10months, 4 year(100cm) and 3 year. Her investigations showed normal hemogram, liver, renal and thyroid functions. Hormonal profile: LH <0.1mIU/ml, FSH <0.1 mIU/ml, Testosterone 0.334ng/ml, DHEAS 17.97µg/dl, Estradiol > 1000pg/ml. CT pelvis showed a heterogeneously enhancing abdomino-pelvic mass with non visualization of ovaries separately suggestive of ovarian mass. She underwent resection of right tuboovarian mass which was suggestive of juvenile granulose cell tumor. Repeat hormonal profile done showed markedly reduced Estradiol levels 17pg/ml. At one year follow-up, there was no vaginal bleeding so far.

### Case 2

6 year old girl presented as precocious puberty with progressive bilateral breast enlargement over 7 months, cyclical vaginal bleeding (3/25-30days) for 4 months and pubic hair development for 2 months. She had a recent height gain over last 1 year. Her past history was unremarkable. Her general examination and vitals were normal. Tanners staging was B3P3. There was no axillary hair or genital ambiguity. Systemic examination was normal. Her investigations showed normal hemogram, liver, renal and thyroid functions. Her bone age, height age and chorological age were respectively 7.8yr, 7 year (119cm) and 6 year. Hormonal profile: LH <0.1mIU/ml, FSH <0.1mIU/ml, Testosterone 0.334ng/ml, DHEAS 17.9µg/dl, Estradiol > 162.8pg/ml. CT pelvis showed a bulky left ovary of size 5.4cm predominantly solid with follicles seen at periphery – possibility of ovarian tumor and pubertal proportion of uterus. She underwent laparascopic resection of right tuboovarian mass which was suggestive of mixed germ cell sex cord stromal tumor. Post-operatively, repeat hormonal profile showed Estradiol levels 14.37pg/ml. At 6 months follow up, there was no vaginal bleeding so far.

## Conclusions

These 2 case reports of ovarian tumor presenting as sexual precocity gives an idea about the heterogeneity of presentation, biochemistry and histopathology. First child had a irregular vaginal bleeding with Estradiol levels of >1000pg/ml due to juvenile granulosa cell tumor. Second had a fairly regular cyclical vaginal bleeding with Estradiol levels of 162.9 pg/ml due to mixed sex cord stromal tumor. Both have successful outcome at 12 months and 6 months respectively of follow up without recurrence.

